# Effect of smoking cessation on new-onset diabetes mellitus in dyslipidemic individuals: A population-based cohort study

**DOI:** 10.18332/tid/205418

**Published:** 2025-06-23

**Authors:** Wooin Seo, Se Young Jung, KeeHyuck Lee, Woo Kyung Bae, Jong Soo Han, Hyejin Lee, Ji Soo Kim, Hye Yeon Koo, Seung Yeon Lee, Kiheon Lee

**Affiliations:** 1 Office of Hospital Information, Seoul National University Bundang Hospital, Seongnam, South Korea; 2 Department of Family Medicine, Seoul National University Bundang Hospital, Seongnam, South Korea; 3 Department of Family Medicine, School of Medicine, Seoul National University, Seoul, South Korea

**Keywords:** smoking behavior change, new-onset diabetes mellitus, dyslipidemia, retrospective cohort study

## Abstract

**INTRODUCTION:**

Smoking is a modifiable risk factor for diabetes mellitus, but the association between changes in smoking behavior and new-onset diabetes mellitus (NODM) in dyslipidemic patients remains unclear. This study aimed to examine how changes in smoking habits affect NODM risk among individuals with dyslipidemia.

**METHODS:**

This retrospective cohort study used data from the Korean National Health Insurance Service–National Sample Cohort (NHIS–NSC). A total of 34282 patients diagnosed with dyslipidemia between 2012 and 2014 were followed until December 2019 (median follow-up: 5 years). Smoking behavior change was defined by transitions in smoking status and intensity across two health examinations. NODM was identified by fasting plasma glucose ≥126 mg/dL or ICD-10 codes E11–E14 with antidiabetic medication.

**RESULTS:**

During follow-up, 2479 participants (7.23%) developed NODM. Those with NODM had higher prevalence of obesity, abdominal obesity, hypertension, abnormal liver function, and family history of diabetes. Current smokers had increased NODM risk (hazard ratio, HR=1.36; 95% CI: 1.22–1.50) versus non-smokers. Heavy smokers had higher risk (HR=1.43; 95% CI: 1.24–1.60) than moderate smokers (HR=1.35; 95% CI: 1.16–1.60). Compared to continuous smokers, quitters had reduced risk (HR=0.79; 95% CI: 0.64–0.98), while reducers showed no significant risk reduction (HR=0.82; 95% CI: 0.63–1.08).

**CONCLUSIONS:**

Among patients with dyslipidemia, smoking cessation was associated with a lower risk of NODM compared to continued smoking. These results suggest potential benefits of quitting smoking in reducing diabetes risk in this population.

## INTRODUCTION

The worldwide incidence of diabetes mellitus in 2021 was 537 million and is anticipated to reach 643 million by 2030^1^. Type 2 Diabetes Mellitus (T2DM) is the most common type of diabetes, accounting for over 90% of all diabetes worldwide. In South Korea, T2DM is a main contributor to disease burden, and its proportion continues to rise^2^. According to the progressive nature of metabolic syndromes (MetS), diabetes is classified as the most severe stage in the evolution of MetS, representing a cluster of complex conditions that cause end-organ damage^3^. In this respect, early detection of high-risk individuals and intervention focusing on target population are cost-effective strategies for diabetes prevention^4^.

Individuals with dyslipidemia are predisposed to T2DM^5^. Components of diagnostic criteria for dyslipidemia, including high triglycerides (TGs) and low plasma high-density lipoprotein cholesterol (HDL-C), were classified as common traits of MetS that may lead to T2DM^3,6^. They were also linked to disturbed glucose metabolism and increased risk of T2DM^7^. Therefore, assessing diabetes risk in dyslipidemic patients and preventing new-onset diabetes mellitus (NODM) through modifiable factors is important.

Smoking is an established risk factor for T2DM^8^. Also, it poses an adverse effect on patients with high cholesterol levels, by increasing low-density lipoprotein cholesterol (LDL-C) and TGs and decreasing HDL-C^9^. In hypercholesterolemic individuals, smoking was one of the risk factors in an association between statin use and incident diabetes^10,11^. Therefore, smoking behavior change should be a primary intervention for preventing T2DM^12^. Although smoking cessation was initially associated with weight gain, potentially elevating the short-term risk of T2DM, this risk gradually decreased over time^13^. While the association between dyslipidemia and T2DM has been widely discussed in previous research, smoking status has been included as a covariate rather than a primary exposure (Supplementary file Table 1). We therefore analyzed longitudinal data from National Health Insurance Service-National Sample Cohort (NHIS-NSC) to investigate the risk factors for NODM among dyslipidemic patients and examine the effect of smoking behavior change on NODM.

## METHODS

### Data source and study setting

We conducted a retrospective cohort study using longitudinal data from the NHIS-NSC, a nationally representative cohort comprising 2.2% of the South Korean population. The NHIS covers nearly 97% of the South Korean population, excluding 3% of Medicaid beneficiaries^14^. The cohort, sampled between 2002 and 2003, included one million individuals stratified to reflect Korea’s demographic diversity across age, sex, and income level.

The database contained health information such as insurance claims, health screening records, and mortality data. Diagnosis codes were documented through claims and reimbursement processes, using the International Classification of Diseases 10th revision (ICD-10) codes. The biennial National Health Screening Program facilitated information on lifestyles and behaviors (alcohol consumption, smoking, and exercise), anthropometric measurements (height, weight, body mass index, and blood pressure) and laboratory test results^15^.

The study protocol was approved by the Institutional Review Board of Seoul National University Bundang Hospital (X-2404-892-907) and the Health Insurance Review and Assessment Service (NHIS-2024-10-2-109). We followed the Strengthening the Reporting of Observational Studies in Epidemiology (STROBE) reporting guideline.

### Study population

**Figure 1:**
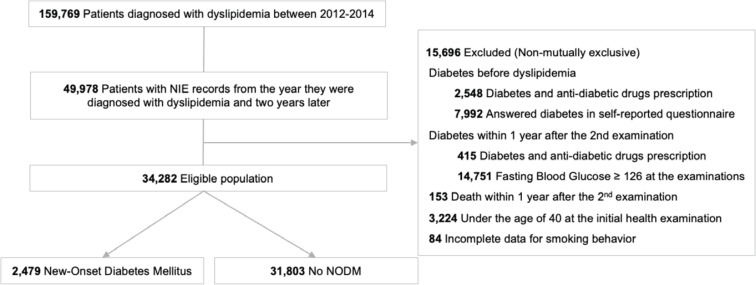
Flow chart of patients according to inclusion and exclusion criteria

This study examined how changes in smoking behavior, particularly smoking cessation, during the two years following a diagnosis of dyslipidemia influenced the risk of NODM over a 5-year observation period. In this sense, we identified patients diagnosed with dyslipidemia at least once between 2012 and 2014 (n=159769). If a patient was diagnosed with dyslipidemia more than once during this period, the diagnosis date was determined based on the first recorded instance. Among dyslipidemic patients, we selected participants who had undergone the health examinations from the year of diagnosis (1st examination) through two subsequent years (n=49978) (2nd examination). To identify the NODM in the study population, we excluded participants who met the following criteria: 1) Those who with pre-existing diabetes before dyslipidemia diagnosis; 2) Patients diagnosed with diabetes within 1 year after the second health examination; 3) Individuals who died within 1 year after the second health examination; 4) Participants aged <40 years at the initial examination; and 5) Participants with incomplete smoking behavior data. A total of 34282 individuals were included for analysis (Figure 1).

### Definitions

*Dyslipidemia*

Dyslipidemia was defined using ICD-10 code E78, confirmed by a physician^16^. In dyslipidemia management, clear and practical knowledge regarding the risk factors, screening, and diagnostic tools are important, thus we aimed to measure changes in patients’ smoking behavior from the time of physician diagnosis of dyslipidemia^17^. To include mild dyslipidemia, patients not taking lipid-lowering medications were also included.

*Changes in smoking behavior*

Current smoking status was surveyed in both the first and second health examination, and categorized into three groups (non-smoker, former smoker, and current smoker). Current smokers additionally reported their daily cigarette consumption. Smoking status was classified based on cigarettes smoked per day: light (<10), moderate (10–19), and heavy (≥20)^18^. Changes in smoking behavior were categorized into four groups^19^: 1) Continuous smokers, participants who maintained or increased smoking intensity between the first and second examinations; 2) Reducers, decreased smoking intensity; 3) Quitters, completely stopped smoking; and 4) Non-smokers, reported as either never smoking or former smokers during both examinations. We defined the changes in smoking as a categorical transition in smoking intensity between two health examinations (i.e. heavy smoking to light smoking; light smoking to mild smoking).

### Covariates

Covariate information was collected on the day of and during the month following the second examination. Including age and sex, individual demographic information was available. Household income was categorized into three groups based on insurance premium. Low-income individuals were defined as insured employees or insured self-employed individuals in the first to third income deciles, and medical aid beneficiaries. Middle-income individuals were defined as individuals in the fourth to seventh income deciles, and high-income individuals were those in the eighth to tenth income deciles. Residential areas were divided into urban and rural. Urban included the capital city (Seoul) and other metropolitans in South Korea.

Obese was defined as having a body mass index (BMI) ≥25 kg/m^2^ and overweight as having a BMI ≥23 kg/m^2^, according to the guidelines from the Korean Society for the Study of Obesity^20^. A waist circumference of ≥90 cm in men and ≥85 cm in women was defined as abdominal obesity. We assessed individual comorbidity status using the Charlson comorbidity index (CCI), based on diagnosis records from the year of the second health examination^21^. Alcohol consumption was classified into three categories (none, moderate, and heavy). According to the National Institute on Alcohol Abuse and Alcoholism (NIAAA) criteria, heavy drinking was defined as consuming more than 4 drinks (56 g of alcohol) per day or 14 drinks (196 g) per week for males, and more than 3 drinks (42 g) per day or 7 drinks (98 g) per week for females^22^. The level of physical activity was calculated based on the metabolic equivalents of tasks (MetS). As the achievement of 500 MetS and more was recommended by the public health guideline, we defined a healthy level of physical activity as 500 MetS and more^23^.

Hypertension was defined as high systolic blood pressure (≥140 mmHg), high diastolic blood pressure (≥90 mmHg), or a diagnosis of hypertension (ICD-10 codes I10-I13 and I15) with antihypertensive medication^24^. Abnormal liver function was defined by the value of serum glutamic oxaloacetic transaminase (SGOT) ≥40 (IU/L) or serum glutamic pyruvate transaminase (SGPT) ≥40 (IU/L)^25^. In addition, analyses were conducted by controlling potential confounding variables such as serum total cholesterol level, fasting plasma glucose, and family history of diabetes. Since some of our study participants used statin therapy that may increase the risk for NODM, we controlled for potential confounding effects of statins on NODM by considering the duration of statin therapy. We extracted patients’ statin prescription records from the day of the second health examination to the last day of observation and calculated the total number of days of statin administration for each patient.

### Study outcomes and follow-up

The primary outcome of this study was NODM in patients with dyslipidemia. Diabetes mellitus was determined when fasting plasma glucose was ≥126 mg/dL or a diagnosis of type 2 diabetes mellitus (ICD-10 codes E11-E14) with antidiabetic medication record^24^. We utilized claim data and health examination records from one year after the second examination. For those who developed NODM during the study, the observation period ended on the date of their first recorded diagnosis or the health examination. For patients without NODM events, the endpoint was either death or the end of the study period (31 December 2019), whichever occurred first.

### Statistical analysis

Descriptive statistics were reported as mean and standard deviation (SD) for continuous variables, and frequencies and percentages for categorical variables. The individual characteristics of the entire study population were presented according to the incidence of NODM and the changes in smoking behavior. We assessed the association between NODM and changes in smoking behavior by estimating NODM-free survival rates using the Kaplan-Meier method and comparing survival rates across different smoking groups with the log-rank test.

The Cox proportional hazards model estimated hazard ratio (HR) and 95% confidence intervals (CIs) values for predicting NODM. We estimated the hazard ratio of current smoking, smoking intensity and changes in smoking behavior. Current smoking status and its intensity were the measurements from the second health examination. All models were adjusted for multiple variables, including demographic factors (age, sex, household income, residential area), anthropometric measures (obesity, abdominal obesity), clinical characteristics [Charlson comorbidity index (CCI) score, hypertension, abnormal liver function], family history of T2DM, lifestyle factors (alcohol consumption, physical activity), metabolic parameters (total cholesterol level, fasting plasma glucose), and duration of statin therapy. These factors were included based on previous literature and clinical judgment, as they are known to be potential confounders affecting the association between smoking and diabetes. We calculated E-values to assess the potential impact of unmeasured confounding on the observed protective effect of smoking cessation on NODM. Larger E-values indicate that stronger unmeasured confounding would be needed to explain away the observed association^26^.

Given the minimal amount of missing data, no imputations were performed (Supplementary file Table 2)^27^. Descriptive statistics were analyzed using the *tableone* package, and survival analysis was performed using *survival*, *survminer*, and *forester* packages in R version 4.3.0. Statistical significance was defined as a two-sided p<0.05.

## RESULTS

Over a median follow-up of 5 years from the second health examination, 2479 (7.23%) were newly diagnosed with diabetes mellitus among the 34282 eligible participants (Table 1). The mean age of the NODM population was 58.88 years (SD=9.53), and 56.1% were male. At the second examination, the proportion of current smokers (20.1%) was significantly higher among NODM group than among non-NODM group (12.7%). The NODM group showed significantly higher proportions of obesity (59.3% vs 39.7%), abdominal obesity (41.9% vs 26.9%), hypertension (26.2% vs 18.1%), abnormal liver function (41.9% vs 26.9%), and family history of T2DM (11.7% vs 8.1%) compared to the non-NODM group (Table 1).

**Table 1:** Baseline characteristics of the study population at first health examination, stratified by NODM incidence (N=34282)

*Characteristics*	*Total* *n (%)*	*Non-NODM* *n (%)*	*NODM* *n (%)*	*p*
**Total**	34282 (100)	31803 (92.7)	2479 (7.2)	
**Age** (years)				0.033
Mean (SD)	59.33 (9.67)	59.37 (9.68)	58.88 (9.53)	0.015
40–49	5586 (16.3)	5168 (16.3)	418 (16.9)	
50–59	12316 (35.9)	11370 (35.8)	946 (38.2)	
60–69	10425 (30.4)	9711 (30.5)	714 (28.8)	
≥70	5955 (17.4)	5554 (17.5)	401 (16.2)	
**Sex**				<0.001
Female	19443 (56.7)	18355 (57.7)	1088 (43.9)	
Male	14839 (43.3)	13448 (42.3)	1391 (56.1)	
**Residence**				0.336
Urban	16021 (46.7)	14886 (46.8)	1135 (45.8)	
Rural	18807 (53.3)	16917 (53.2)	1344 (54.2)	
**Income level**				0.294
Low	8157 (24.0)	7554 (23.9)	603 (24.5)	
Middle	10710 (31.5)	9913 (31.4)	797 (32.4)	
High	15143 (44.5)	14086 (44.6)	1057 (43.0)	
**Charlson comorbidity index,** mean (SD)	0.21 (0.48)	0.20 (0.48)	0.22 (0.50)	0.079
**Smoking**				<0.001
None	29766 (86.8)	27784 (87.4)	1982 (80.0)	
Light	649 (1.9)	593 (1.9)	56 (2.3)	
Moderate	1802 (5.3)	1607 (5.1)	195 (7.9)	
Heavy	2065 (6.0)	1819 (5.7)	246 (9.9)	
**Alcohol consumption**				<0.001
None	21921 (64.0)	20533 (64.9)	1388 (56.0)	
Moderate	3444 (10.0)	3213 (10.1)	231 (9.3)	
Heavy	8908 (26.0)	8048 (25.3)	860 (34.7)	
**Physical activity** (MetS)				0.073
<500	17144 (50.0)	15862 (49.9)	1282 (51.8)	
≥500	17144 (50.0)	15941 (50.1)	1197 (48.2)	
**BMI** (kg/m^2^)				<0.001
Underweight (<23)	10880 (31.7)	10441 (32.8)	439 (17.7)	
Overweight (≥23)	9314 (27.2)	8743 (27.5)	571 (23.0)	
Obese (≥25)	14088 (41.1)	12619 (39.7)	1469 (59.3)	
Mean (SD)	24.47 (3.06)	24.37 (3.03)	25.77 (3.17)	<0.001
**Abdominal obesity**	9586 (28.0)	8548 (26.9)	1038 (41.9)	<0.001
**Waist circumference** (cm), mean (SD)	82.39 (8.51)	82.09 (8.46)	86.23 (8.22)	<0.001
**Hypertension**	6417 (18.7)	5767 (18.1)	650 (26.2)	<0.001
**Abnormal liver function**	5192 (15.1)	4533 (14.3)	659 (26.6)	<0.001
**Statin use**	5070 (14.8)	4532 (14.3)	538 (21.7)	<0.001
**Family history of T2DM**				<0.001
No	19646 (57.3)	18284 (57.5)	1362 (54.9)	
Unknown	11785 (34.4)	10957 (34.5)	828 (33.4)	
Yes	2851 (8.3)	2562 (8.1)	289 (11.7)	
**Clinical measurements**				
Systolic BP (mmHg), mean (SD)	124.78 (14.28)	124.54 (14.28)	127.83 (13.91)	<0.001
Diastolic BP (mmHg), mean (SD)	77.08 (9.54)	76.93 (9.52)	79.04 (9.59)	<0.001
FPG (mg/dL), mean (SD)	96.30 (11.02)	95.54 (10.59)	106.05 (11.69)	<0.001
Total cholesterol (mg/dL), mean (SD)	204.47 (45.77)	204.62 (45.79)	202.65 (45.44)	0.040
TGs (mg/dL), mean (SD)	147.23 (117.07)	144.31 (114.42)	184.75 (141.70)	<0.001
HDL-C (mg/dL), mean (SD)	54.92 (27.00)	55.20 (27.73)	51.31 (14.32)	<0.001
LDL-C (mg/dL), mean (SD)	121.35 (45.15)	121.71 (45.39)	116.73 (41.65)	<0.001
SGOT (U/L), mean (SD)	27.39 (20.89)	27.18 (21.19)	30.05 (16.22)	<0.001
SGPT (U/L), mean (SD)	26.49 (26.06)	26.01 (26.24)	32.63 (22.69)	<0.001

NODM: new-onset diabetes mellitus. T2DM: type 2 diabetes mellitus. BMI: body mass index. MetS: metabolic equivalents of task. FPG: fasting plasma glucose. BP: blood pressure. LDL-C: low-density lipoprotein cholesterol. TGs: triglycerides. HDL-C: high-density lipoprotein cholesterol. SGOT: serum glutamic oxaloacetic transaminase. SGPT: serum glutamic pyruvate transaminase.

Baseline characteristics differed across the groups based on changes in smoking behavior (Table 2). Quitters tended to be older (mean age=60.16 years, SD=9.54), more obese and have chronic diseases (hypertension and abnormal liver function). Due to the very low smoking rates among women, prevalence of male population was higher in all smoker groups, while most women were non-smokers. Among those who reported smoking on the first examination, regardless of smoking intensity, >60% of them continued their smoking, while >30% either reduced their smoking or quit (Supplementary file Table 3). The duration of statin use among dyslipidemic patients who received statin therapy was similar across smoking groups. Consistently across all groups, atorvastatin was the most frequently prescribed type of statin, followed by rosuvastatin and simvastatin (Supplementary file Table 4).

**Table 2:** Baseline characteristics at first health examination according to changes in smoking behavior (N=34282)

*Characteristics*	*Continuous smoker* *n (%)*	*Reducer* *n (%)*	*Quitter* *n (%)*	*Non-smoker* *n (%)*	*p*
**Patients,** n	3911	605	1116	28650	
**Patients with NODM**	436 (11.1)	61 (10.1)	110 (9.9)	1872 (6.5)	<0.001
**Age** (years)					<0.001
Mean (SD)	54.54 (8.92)	56.31 (9.70)	56.60 (9.69)	60.16 (9.54)	<0.001
40–49	1304 (33.3)	171 (28.3)	292 (26.2)	3819 (13.3)	
50–59	1502 (38.4)	224 (37.0)	413 (37.0)	10177 (35.5)	
60–69	823 (21.0)	147 (24.3)	280 (25.1)	9175 (32.0)	
≥70	282 (7.2)	63 (10.4)	131 (11.7)	5479 (19.1)	
**Sex**					<0.001
Female	340 (8.7)	76 (12.6)	165 (14.8)	18862 (65.8)	
Male	3571 (91.3)	529 (87.4)	951 (85.2)	9788 (34.2)	
**Charlson comorbidity index,** mean (SD)	0.16 (0.42)	0.14 (0.37)	0.18 (0.43)	0.21 (0.49)	<0.001
**BMI** (kg/m^2^)					<0.001
Underweight (<23)	1173 (30.0)	179 (29.6)	281 (25.2)	9247 (32.3)	
Overweight (≥23)	1025 (26.2)	168 (27.8)	286 (25.6)	7835 (27.3)	
Obese (≥25)	1713 (43.8)	258 (42.6)	549 (49.2)	11568 (40.4)	
**Abdominal obesity**	1155 (29.5)	174 (28.8)	365 (32.7)	7892 (27.6)	<0.001
**Hypertension**	656 (16.8)	105 (17.4)	224 (20.1)	5432 (19.0)	0.764
**Abnormal liver function**	926 (23.7)	134 (22.1)	284 (25.4)	3848 (13.4)	<0.001
**Smoking** (1st health examination)					<0.001
None	620 (15.9)	0 (0)	0 (0)	28650 (100)	
Light	412 (10.5)	0 (0)	235 (21.1)	0 (0)	
Moderate	1323 (33.8)	172 (28.4)	452 (40.5)	0 (0)	
Heavy	1556 (39.8)	433 (71.6)	429 (38.4)	0 (0)	
**Smoking** (2nd health examination)					<0.001
None	0 (0)	0 (0)	1116 (100)	28650 (100)	
Light	435 (11.1)	214 (35.4)	0 (0)	0 (0)	
Moderate	1411 (36.1)	391 (64.6)	0 (0)	0 (0)	
Heavy	2065 (52.8)	0 (0)	0 (0)	0 (0)	
**Statin use**	632 (16.2)	99 (16.4)	227 (20.3)	4112 (14.4)	<0.001
**Family history of T2DM**					0.260
No	2314 (59.2)	336 (55.5)	644 (57.7)	16352 (57.1)	
Unknown	1273 (32.5)	219 (36.2)	393 (35.2)	9900 (34.6)	
Yes	324 (8.3)	50 (8.3)	79 (7.1)	2398 (8.4)	
**FPG** (mg/dL), mean (SD)	96.85 (11.32)	98.00 (11.45)	97.86 (11.44)	96.12 (10.94)	0.190

NODM: new-onset diabetes mellitus. BMI: body mass index. T2DM: type 2 diabetes mellitus. FPG: fasting plasma glucose.

**Figure 2:**
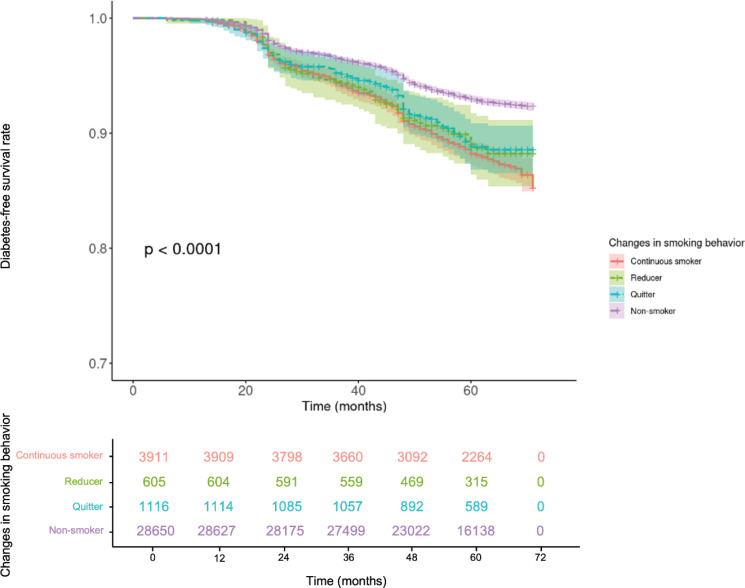
Kaplan-Meier curves showing the incidence of NODM stratified by smoking behavior changes (N=34282)

**Figure 3:**
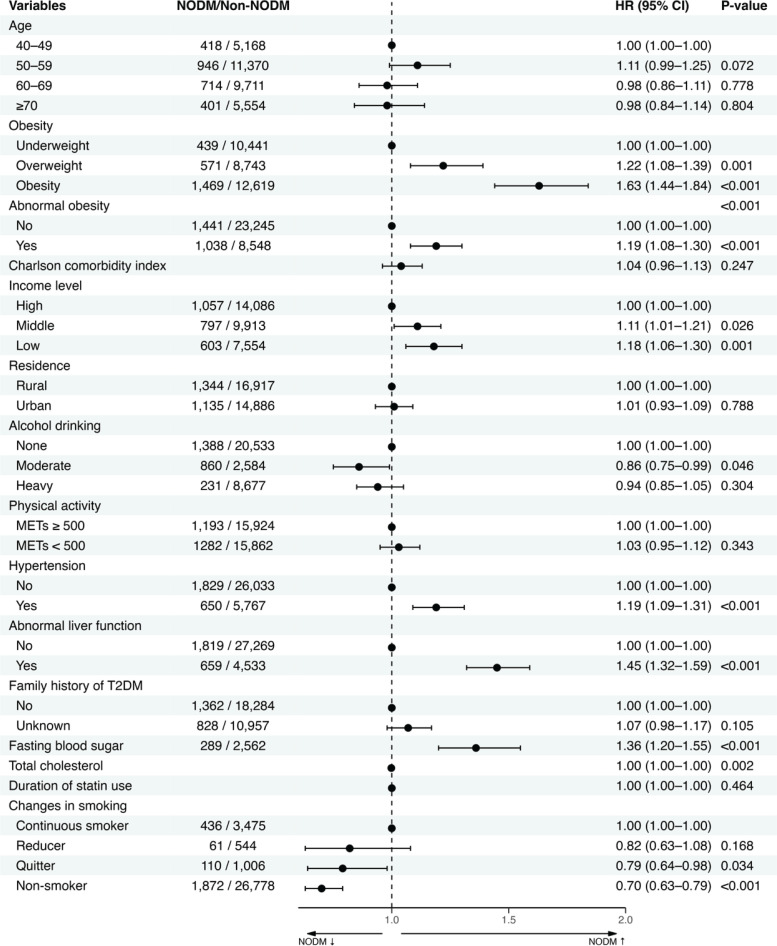
Forest plot of risk factors for new-onset diabetes mellitus in patients with dyslipidemia (N=33971)

The proportional hazards (PH) assumption was tested using the Schoenfeld residual test. A violation of the PH assumption was observed for sex variable; therefore, sex was included as a stratification factor in the Cox model to allow for different baseline hazard functions by sex. Current smokers at the time of second examination had a significantly increased risk of NODM, with a HR of 1.36 (95% CI: 1.22–1.50), compared to non-smokers (Supplementary file Table 5). They had different HR based on smoking intensity: heavy smokers (HR=1.43; 95% CI: 1.24–1.60), and moderate smokers (HR=1.35; 95% CI:1.16–1.60). Light smokers did not show a statistically significant increase in NODM risk (HR=1.15; 95% CI: 0.87–1.50) (Supplementary file Table 5). In the main analysis, we assessed the association between changes in smoking behavior and NODM risk. Kaplan-Meier survival curves revealed significant differences in NODM incidence rates between groups (p<0.001), with non-smokers and quitters exhibiting the lowest risk of NODM (Figure 2). However, the significance disappeared when analyzed in females (p=0.32) (Supplementary file Figure 1). After adjusting for confounders, non-smokers demonstrated a significantly lower risk of NODM compared to continuous smokers (HR=0.70; CIs: 0.63–0.79). Quitters also showed a significant reduction in risk (HR=0.79, 95% CI: 0.64–0.98), whereas reducers did not reach statistical significance (HR=0.82; 95% CI: 0.63–1.08) (Figure 3). Several variables were identified as risk factors for NODM. These included obesity (HR=1.63; 95% CI: 1.44–1.84), abdominal obesity (HR=1.19; 95% CI: 1.08–1.30), lower household income (HR=1.18; 95% CI: 1.06–1.30), hypertension (HR=1.19; 95% CI: 1.09–1.31), abnormal liver function (HR=1.45; 95% CI: 1.32–1.59), family history of diabetes (HR=1.36; 95% CI: 1.20–1.55), and elevated fasting plasma glucose levels (HR=1.08; 95% CI: 1.08–1.09) (Figure 3; and Supplementary file Table 6).

## DISCUSSION

To our knowledge, this is the first large-scale, longitudinal study to investigate the effect of smoking cessation on NODM risk among dyslipidemic patients. Using a nationally representative sample cohort, we were able to utilize a larger dyslipidemic patient cohort compared to previous studies of small samples from specific hospitals^28,29^. Among 34282 patients diagnosed with dyslipidemia between 2012 and 2014, 2479 patients (7.23%) developed NODM during the 5-year observation period. This incidence was higher than previous research with a comparable median follow-up period, which reported a 3.2% NODM rate in the general Korean population^30^. This difference may be attributed to our study population’s predisposition to T2DM due to their dyslipidemia^5,7^.

Smoking increases the risk of T2DM by inducing insulin resistance or decreased insulin secretion through oxidative stress, inflammation and endothelial dysfunction^31^. Among patients diagnosed with dyslipidemia, current smokers had a 36% higher risk of NODM compared to non-smokers. Specifically, moderate and heavy smoking levels were associated with 35% and 43% higher risks of NODM, respectively. This dose-response relationship between current smoking and the risk of diabetes is consistent with findings from previous studies^8^. We also measured changes in smoking status using longitudinal data. For quitters, the risk of NODM was significantly decreased with a HR of 0.79, compared to continuous smokers. This risk reduction was more pronounced than that observed in quitters from general Korean population (HR=0.85; 95% CI: 0.83–0.87)^30^. Despite similar incidence rates of NODM between reducers (10.1%) and quitters (9.9%), reducers showed no significant decreased risk of NODM (HR=0.82; 95% CI: 0.63–1.08). The wide confidence intervals observed in reducers likely derived from two possible reasons. They comprised only 10.7% (605 of 5632) of total current smokers, and a majority (64.6%) were moderate smokers – a group identified at risk for NODM in our study. According to previous studies, risk reduction of smoking cessation was time-dependent, with the highest HR observed among the most recent quitters and lower HR among earlier quitters in the long-term^13^. In this study, long-term quitters were classified as non-smokers since they reported ‘not smoking’ in both health examinations. Consequently, our study’s definition of quitters was limited to recent cessation, and the definition of non-smokers did not account for the potential long-term effects of smoking cessation, which may represent a limitation of our research. This suggests the need for future research to differentiate between the effects of lifelong non-smoking and long-term smoking cessation.

Since T2DM has multiple risk factors, we identified baseline risk factors of dyslipidemic patients and included them in the model. In the multivariable Cox regression model, several covariates such as male gender, obesity, and abdominal obesity significantly increased the risk of NODM. Previous research showed conflicting evidence regarding the relationship between alcohol consumption and diabetes mellitus^32^. In this study, alcohol consumption was not significantly associated with NODM.

In other studies, smoking cessation initially increased the short-term risk of NODM, but this risk decreased progressively when smoking cessation was sustained for more than 5 years^20^. One possible explanation for these findings was weight gain and the increase in waist circumference following smoking cessation. However, in our study population of patients diagnosed with dyslipidemia, smoking cessation was associated with a decreased risk of NODM within a 5-year period. This difference may be attributed to the baseline characteristics of our study participants, who were already at higher risk of weight-related complications. At the time of dyslipidemia diagnosis, over 40% of participants were obese, and nearly 70% were either obese or overweight. Therefore, for patients with dyslipidemia, smoking cessation may offer more significant diabetes-related health benefits compared to the general population. Nevertheless, given that previous smoking cessation studies established observation periods of 10 years^8^, a more extended observation period is required to investigate the long-term effects of changes in smoking behavior.

### Limitations

There were some limitations in this study. First, the generalizability of our findings should be validated by using data from diverse ethnic and cultural populations. Since the study participants were limited to the Korean population, the results cannot be generalized to other ethnic groups with different T2DM prevalence and incidence rates. Also, the effect of smoking in this study was largely confined to the male population. In the Kaplan-Meier estimates in Supplementary file Figure 1, significant differences in incidence rates of T2DM were observed only among males, which contributed to the overall population effect. Our study population was subject to a potential bias due to the under-reporting trend and subsequent underestimation of female smokers. In fact, 97.9% of females in our study identified themselves as non-smokers. One study revealed that the ratio of cotinine-verified to self-reported smoking rates was 2.36 for women in South Korea^33^.

Second, this study has limitations in the assessment of smoking status. Notably, smoking abstinence was not biochemically verified, which may have led to misclassification. One research has shown that more than 30% of individuals who successfully quit smoking for one year ultimately relapsed within the subsequent decade^34^. Furthermore, information on use of alternative tobacco products was not collected. Since emerging nicotine products such as electronic nicotine delivery systems (ENDS or e-cigarettes) and heated tobacco products (HTPs) were popular in the transition state to tobacco cessation^35,36^, we cannot exclude the possibility of exposure to these products among our study population during the observation period. Third, our measurement of statin use duration relied on prescription dates, which may not accurately reflect actual patient adherence. The duration of statin use and the primary statin type based on prescription data were not significantly different across smoking groups, suggesting no noticeable bias related to statin use in our study (Supplementary file Table 2). Last but not least, several potential confounders were not observed in this study, including dietary factors and sedentary lifestyle. The E-value calculated from the hazard ratio of quitters was 1.83 (upper confidence limit: 1.16), suggesting that an unmeasured confounder of this magnitude could potentially explain away the observed association.

## CONCLUSIONS

The results from this 5-year longitudinal study suggest that smoking cessation was associated with decreased risk of NODM among dyslipidemic patients. These findings strengthen the evidence on the association between smoking and T2DM, particularly in individuals with metabolic syndrome who have complex and overlapped risk factors for NODM.

## Supplementary Material



## Data Availability

The data will be made available upon request and approval of a proposal by Korean National Health Insurance Service (https://nhiss.nhis.or.kr/).
