# Supplementary material for: Effect of smoking cessation on new-onset diabetes mellitus in dyslipidemic individuals: A population-based cohort study
Source: Tob Induc Dis. 2025 Jun 23;23:10.18332/tid/205418. doi: 10.18332/tid/205418 (PMC12184095; doi:10.18332/tid/205418)
Supplement: Supplementary file 1 [file TID-23-82-s1.pdf]

Supplementary Table 1. Previous Research on Dyslipidemia and NODM

Author	year	Country	Exposure variables	Study design	Study setting	Number of participants	Outcome	Main finding
**Original Investigation**								
**Jacobs et al.[1]**	2005	US	Diabetes Mellitus	Cross-sectional	Population-Based	498 participants	Dyslipidemia	Many persons with diabetes remain uncontrolled for dyslipidemia. Intensified efforts at screening and treatment according to current guidelines are warranted. Less than one-third of men and only one-fifth of women with diabetes are in control for LDL-C, defined as <2.6mmol/l (<100mg/dl); over 70% are not at goal.
**Seo et al.[2]**	2011	South Korea	Lipid profiles	Cohort	Population-Based	5,577 participants	NODM	The ratio of TC to HDL and apoB to HDL showed a significant association with increased risk of type 2 diabetes, compared with other lipoprotein parameters.
**Lee et al.[3]**	2012	Taiwan	Dyslipidemia treated with fibrates	Cohort	Population-Based	3,815 dyslipidaemic patients	NODM	The risk estimates for NODM for users of fenofibrate (HR 1.30; 95% CI 0.82, 2.05) and gemfibrozil (HR 0.771; 95% CI 0.49, 1.22) were not associated with an increased risk of developing NODM (P > 0.05).
**Rhee et al.[4]**	2017	South Korea	Large variation in total cholesterol levels	Cohort	Population-Based	2,827,950 participants	NODM	During the follow-up period, 3.4% of participants developed diabetes. The hazard ratio (HR) for diabetes onset exceeded 1.0 starting from the eighth decile of total cholesterol (TC) variation. After adjusting for confounding variables, the highest decile group demonstrated an elevated risk for diabetes development (HR: 1.139, 95% CI: 1.116-1.160).
**Roy et al.[5]**	2019	India	Dyslipidemia	Cohort	Hospital-Based	270 disylipidemic patients	NODM	Out of 270 dyslipidemic patients, 19 patients developed statin-induced

								new onset of diabetes and 69 were classified as pre-diabetic.<br>The major risk factors were: dose, gender, age, geriatric patients, and duration of the therapy.
**Kim et al.**[6]	2019	South Korea	Statin use	Cohort	Population-Based	38,502 participants	NODM	The risk of NODM was not associated with an increase in the cumulative duration of statin use or with non-recent use. Only recent short-term use of statin was associated with an increased risk of NODM. Diabetes screen-ing are warranted during initial statin therapy.
**Kim et al.**[7]	2020	South Korea	Statin use	Cohort	Population-Based	21,469 hypercholesterolemic patients	NODM	Statin users demonstrated a significantly higher risk of new-onset diabetes mellitus (NODM) compared to non-users.<br>After adjusting for confounding factors including age and lifestyle variables, the adjusted hazard ratios (aHRs) were higher in women (aHR: 1.86, 95% CI: 1.66-2.10) than in men (aHR: 1.43, 95% CI: 1.31-1.57).
**Peng et al.**[8]	2021	China	Dyslipidemia	Cohort	Population-Based	7,329 subjects	NODM	Over a mean follow-up period of 3.4 years, 387 participants (5.28%) developed new-onset Type 2 diabetes mellitus (T2DM).<br>Compared to those with normal lipid profiles, participants with hypercholesterolemia, hypertriglyceridemia, and low high-density lipoprotein cholesterol (HDL-C) showed significantly increased T2DM risk<br>(HR: 1.48, 95% CI: 1.11-1.96; HR: 1.92, 95% CI: 1.49-2.46; and HR:

								1.67, 95% CI: 1.35-2.07, respectively).
**Ahmmed et al.**[9]	2021	Bangladash	T2DM	Cross-sectional	Hospital-Based	132 T2DM patients	Dyslipidemia	The study revealed higher dyslipidemia prevalence in female T2DM patients (75.7%) compared to males (72.6%), with females having 1.74 times higher odds of developing the condition.<br>Several risk factors were significantly associated with dyslipidemia, including early middle age (30-39 years), obesity, increased waist circumference, hypertension, physical inactivity, and tobacco use.

Supplementary Table 2. Missing value

**Variables**	N	%
Age	3	0.008
Sex	1	0.002
Residence	0	0
Income level	272	0.79
Charlson comorbidity index	0	0
Smoking	0	0
Alchol consupmtion	9	0.02
Physical activity	21	0.06
Obesity	0	0
Abdominal obesity	10	0.02
Hypertension	3	0.008
Abnormal liver function	2	0
Statin use	0	0
Family history of T2DM	0	0
BMI	13	0.03
Waist circumference	9	0.02
Systolic BP	5	0.01
Diastolic BP	5	0.01
FPG	2	0.005
Total cholesterol	2	0.005
TGs	2	0.005
HDL-C	2	0.005
LDL-C	185	0.53
SGOT	2	0.005
SGPT	2	0.005

Abbreviations: BMI, body mass index; METs, Metabolic Equivalents of Task; FPG, fasting plasma glucose; BP, blood pressure; LDL-C, low-density lipoprotein cholesterol; TGs, triglycerides; HDL-C, high-density lipoprotein cholesterol; SGOT, Serum Glutamic Oxaloacetic Transaminase; SGPT, Serum Glutamic Pyruvate Transaminase.

Supplementary Table 3. Changes in smoking behavior according to smoking status at the first examination

	None	Light	Moderate	Heavy
Continuous smoker	0(0)	412(63.7)	1323(68.0)	1556(64.4)
Reducer	0(0)	0(0)	172(8.8)	433(17.9)
Quitter	0(0)	235(36.3)	452(23.2)	429(17.7)
Non-smoker	620(2.1)	0(0)	0(0)	0(0)

Supplementary Table 4. Statin user among dyslipidemia patients (n=5,070)

		Continuous smoker	Reducer	Quitter	Non-smoker	p-value
No. of patients (%)		632 (12.5)	99 (2)	227 (4.5)	4112 (81.1)	
Statin type						0.31
	atorvastatin	369 (58.4)	55 (55.6)	129 (56.8)	2378 (57.8)	
	rosuvastatin	170 (26.9)	36 (36.4)	66 (29.1)	1162 (28.3)	
	simvastatin	41 (6.5)	6 (6.1)	20 (8.8)	298 (7.2)	
	fluvastatin	1 (0.2)	0 (0.0)	1 (0.4)	16 (0.4)	
	lovastatin	5 (0.8)	0 (0.0)	0 (0.0)	9 (0.2)	
	pitavastatin	27 (4.3)	0 (0.0)	7 (3.1)	167 (4.1)	
	pravastatin	19 (3.0)	2 (2.0)	4 (1.8)	82 (2.0)	
Duration of statin use, median(IQR)		19.00 (9.00-45.25)	17.00 (9.00-40.00)	22.00 (11.00-46.50)	19.00 (9.00-46.00)	0.38

Supplementary Table 5. Risk of type 2 diabetes mellitus according to baseline smoking status

		NODM/Non-NODM	HR	95% CI	p-value
Smoking status (2nd examination)					
	Current non-smoker	1982/27784	1		
	Current smoker	497/4019	1.36	1.22-1.50	<0.001
Smoking intensity (2nd examination)					
	None	1982/27784	1		
	Light smoker	56/593	1.15	0.87-1.50	0.323
	Moderate smoker	195/1607	1.35	1.16-1.60	<0.001
	Heavy smoker	246/1819	1.43	1.24-1.60	<0.001

Supplementary Table 6. Multivariable Cox proportional hazards regression results of the association between NODM and changes in smoking behavior

		NODM /Non-NODM	HR	95% CI	p-value
Age					
	40-49	418/5,168	1		
	50-59	946/11,370	1.11	0.99-1.25	0.072
	60-69	714/9,711	0.98	0.86-1.11	0.778
	≥ 70	401/5,554	0.98	0.84-1.14	0.804
Obesity					
	Underweight	439/10,441	1		
	Overweight	571/8,743	1.22	1.08-1.39	0.001
	Obesity	1,469/12,619	1.63	1.44-1.84	<0.001
Abnormal obesity					<0.001
	No	1,441/23,245	1		
	Yes	1,038/8,548	1.19	1.08-1.30	
Charlson comorbidity index			1.04	0.96-1.13	0.247
Income level					
	High	1,057/14,086	1		
	Middle	797/9,913	1.11	1.01-1.21	0.026
	Low	603/7,554	1.18	1.06-1.30	0.001
Residence					
	Rural	1,344/16,917	1		
	Urban	1,135/14,886	1.01	0.93-1.09	0.788
Alcohol drinking					
	None	1,388/20,533	1		
	Moderate	860/2,584	0.86	0.75-0.99	0.046
	Heavy	231/8,677	0.94	0.85-1.05	0.304
Physical activity					0.343
	METs ≥ 500	1,193/15,924	1		
	METs < 500	1,282/15,862	1.03	0.95-1.12	
Hypertension					<0.001
	No	1,829/26,033	1		
	Yes	650/5,767	1.19	1.09-1.31	
Abnormal liver function					<0.001
	No	1,819/27,269	1		
	Yes	659/4,533	1.45	1.32-1.59	
Family history of T2DM					
	No	1,362/18,284	1		
	Unknown	828/10,957	1.07	0.98-1.17	0.105
	Yes	289/2,562	1.36	1.20-1.55	<0.001
Fasting blood sugar (mg/dL)			1.08	1.08-1.09	<0.001
Total cholesterol (mg/dL)			0.998	0.997-0.999	0.002

Duration of statin use (months)			1.0001	0.9999-1.0003	0.464
Changes in smoking					
	Continuous smoker	436/3,475	1		
	Reducer	61/544	0.82	0.63-1.08	0.168
	Quitter	110/1,006	0.79	0.64-0.98	0.034
	Non-smoker	1,872/26,778	0.70	0.63-0.79	<0.001
**C-index: 0.77**					
Abbreviations: HR, Hazard Ratio; CIs, confidence intervals; BMI, body mass index; METs, Metabolic Equivalents of Task; T2DM, Type 2 Diabetes Mellitus					

Supplementary Figure 1. Kaplan-Meier curves for New-onset Diabetes Mellitus according to changes in smoking behavior (left: male, right: female)

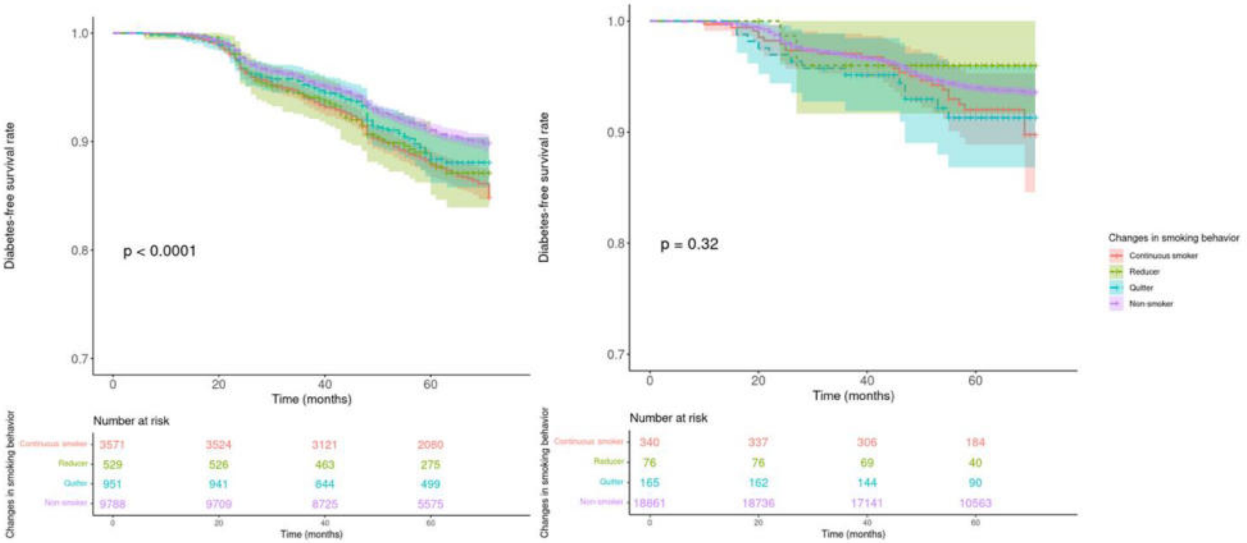

Supplementary References

1. Jacobs MJ, Kleisli T, Pio JR, et al. Prevalence and control of dyslipidemia among persons with diabetes in the United States. Diabetes Res Clin Pract. 2005;70(3):263-269. doi:10.1016/j.diabres.2005.03.032
2. Seo MH, Bae JC, Park SE, et al. Association of lipid and lipoprotein profiles with future development of type 2 diabetes in nondiabetic Korean subjects: a 4-year retrospective, longitudinal study. J Clin Endocrinol Metab. 2011;96(12):E2050-E2054. doi:10.1210/jc.2011-1857
3. Lee CY, Huang KH, Lin CC, Tsai TH, Shih HC. A neutral risk on the development of new-onset diabetes mellitus (NODM) in Taiwanese patients with dyslipidaemia treated with fibrates. ScientificWorldJournal. 2012;2012:392734. doi:10.1100/2012/392734
4. Rhee EJ, Han K, Ko SH, Ko KS, Lee WY. Increased risk for diabetes development in subjects with large variation in total cholesterol levels in 2,827,950 Koreans: A nationwide population-based study. PLoS One. 2017;12(5):e0176615. doi:10.1371/journal.pone.0176615
5. Roy R, Ajithan A, Joseph A, Mateti UV, K S. Statin-induced new onset of diabetes in dyslipidemic patients: a retrospective study. Postgrad Med. 2019;131(6):383-387. doi:10.1080/00325481.2019.1643636
6. Kim DW, Kim DH, Park JH, et al. Association between statin treatment and new-onset diabetes mellitus: a population based case-control study. Diabetol Metab Syndr. 2019;11:30. doi:10.1186/s13098-019-0427-9
7. Kim YS, Han YE, Choi EA, et al. Statin use increased new-onset diabetes in hypercholesterolemic individuals: Data from the Korean National Health Insurance Service-National Health Screening Cohort database (NHIS-HEALS). Prim Care Diabetes. 2020;14(3):246-253. doi:10.1016/j.pcd.2019.08.005
8. Peng J, Zhao F, Yang X, et al. Association between dyslipidemia and risk of type 2 diabetes mellitus in middle-aged and older Chinese adults: a secondary analysis of a nationwide cohort. BMJ Open. 2021;11(5):e042821. doi:10.1136/bmjopen-2020-042821
9. Ahmmed MS, Shuvo SD, Paul DK, et al. Prevalence of dyslipidemia and associated risk factors among newly diagnosed Type-2 Diabetes Mellitus (T2DM) patients in Kushtia, Bangladesh. PLOS Glob Public Health. 2021;1(12):e0000003. doi:10.1371/journal.pgph.0000003

STROBE Statement—Checklist of items that should be included in reports of ***cohort studies***

	Item No	Recommendation	Page No
**Title and abstract**	1	(*a*) Indicate the study's design with a commonly used term in the title or the abstract<br>(*b*) Provide in the abstract an informative and balanced summary of what was done and what was found	1,2
**Introduction**			
Background/rationale	2	Explain the scientific background and rationale for the investigation being reported	2-3
Objectives	3	State specific objectives, including any prespecified hypotheses	3
**Methods**			
Study design	4	Present key elements of study design early in the paper	3
Setting	5	Describe the setting, locations, and relevant dates, including periods of recruitment, exposure, follow-up, and data collection	3
Participants	6	(*a*) Give the eligibility criteria, and the sources and methods of selection of participants. Describe methods of follow-up<br>(*b*) For matched studies, give matching criteria and number of exposed and unexposed	4
Variables	7	Clearly define all outcomes, exposures, predictors, potential confounders, and effect modifiers. Give diagnostic criteria, if applicable	4-5
Data sources/ measurement	8*	For each variable of interest, give sources of data and details of methods of assessment (measurement). Describe comparability of assessment methods if there is more than one group	4-5
Bias	9	Describe any efforts to address potential sources of bias	9
Study size	10	Explain how the study size was arrived at	4
Quantitative variables	11	Explain how quantitative variables were handled in the analyses. If applicable, describe which groupings were chosen and why	4-5
Statistical methods	12	(*a*) Describe all statistical methods, including those used to control for confounding<br>(*b*) Describe any methods used to examine subgroups and interactions<br>(*c*) Explain how missing data were addressed<br>(*d*) If applicable, explain how loss to follow-up was addressed<br>(*e*) Describe any sensitivity analyses	6
**Results**			
Participants	13*	(a) Report numbers of individuals at each stage of study—eg numbers potentially eligible, examined for eligibility, confirmed eligible, included in the study, completing follow-up, and analysed<br>(b) Give reasons for non-participation at each stage	4

		(c) Consider use of a flow diagram	
Descriptive data	14*	(a) Give characteristics of study participants (eg demographic, clinical, social) and information on exposures and potential confounders<br>(b) Indicate number of participants with missing data for each variable of interest<br>(c) Summarise follow-up time (eg, average and total amount)	6-7
Outcome data	15*	Report numbers of outcome events or summary measures over time	6
Main results	16	(*a*) Give unadjusted estimates and, if applicable, confounder-adjusted estimates and their precision (eg, 95% confidence interval). Make clear which confounders were adjusted for and why they were included<br>(*b*) Report category boundaries when continuous variables were categorized<br>(*c*) If relevant, consider translating estimates of relative risk into absolute risk for a meaningful time period	7
Other analyses	17	Report other analyses done—eg analyses of subgroups and interactions, and sensitivity analyses	9
**Discussion**			
Key results	18	Summarise key results with reference to study objectives	8
Limitations	19	Discuss limitations of the study, taking into account sources of potential bias or imprecision. Discuss both direction and magnitude of any potential bias	9
Interpretation	20	Give a cautious overall interpretation of results considering objectives, limitations, multiplicity of analyses, results from similar studies, and other relevant evidence	8
Generalisability	21	Discuss the generalisability (external validity) of the study results	9
**Other information**			
Funding	22	Give the source of funding and the role of the funders for the present study and, if applicable, for the original study on which the present article is based	10

*Give information separately for exposed and unexposed groups.

Note: An Explanation and Elaboration article discusses each checklist item and gives methodological background and published examples of transparent reporting. The STROBE checklist is best used in conjunction with this article (freely available on the Web sites of PLoS Medicine at http://www.plosmedicine.org/, Annals of Internal Medicine at http://www.annals.org/, and Epidemiology at http://www.epidem.com/). Information on the STROBE Initiative is available at http://www.strobe-statement.org.

© 2025 Seo W. et al.
